# Biparatopic targeting of CD38 using CD38-specific trimeric antibody recruiters improves cytotoxicity against multiple myeloma

**DOI:** 10.3389/fimmu.2026.1915205

**Published:** 2026-09-24

**Authors:** Henry Risch, Anna Josephine Gebhardt, Niklas Hagen, Anya Duttmann, Marten Dannenberg, Sofie Kämereit, Ricardo Ahrens, Maximilian Al-Bazaz, Kristoffer Riecken, Boris Fehse, Katja Weisel, Peter Bannas, Friedrich Koch-Nolte, Luca Julius Pape, Julia Hambach

**Affiliations:** 1 Department of Diagnostic and Interventional Radiology and Nuclear Medicine, University Medical Center Hamburg-Eppendorf, Hamburg, Germany; 2 Institute of Immunology, University Medical Center Hamburg-Eppendorf, Hamburg, Germany; 3 Research Department Cell and Gene Therapy, Department of Stem Cell Transplantation, University Medical Center Hamburg-Eppendorf, Hamburg, Germany; 4 Department of Oncology, Hematology and Bone Marrow Transplantation, University Medical Center Hamburg-Eppendorf, Hamburg, Germany; 5 Hamburg Center for Translational Immunology (HCTI), University Medical Center Hamburg-Eppendorf (UKE), Hamburg, Germany; 6 German Center for Child and Adolescent Health (DZKJ), Partner Site Hamburg, Hamburg, Germany; 7 Department of Diagnostic and Interventional Radiology, Albertinen Hospital, Hamburg, Germany

**Keywords:** antibody recruiting molecules, avidity, biparatopic, bispecific engagers, CD38, complement-dependent cytotoxicity, multiple myeloma, nanobodies

## Abstract

**Rationale:**

Antibody recruiters are an innovative class of immunotherapeutics, comprising a tumor-binding module and an antibody-binding module to redirect endogenous antibodies toward target cells. Recruited antibodies can then induce Fc-dependent effector functions to lyse tumor cells. The clinical success of CD38-directed monoclonal antibodies daratumumab and isatuximab has established CD38 as a validated target for the treatment of multiple myeloma (MM). Since daratumumab treatment can result in antigen loss or mutation of the binding site, novel immunotherapeutics should explore different binding mechanisms for maximum efficacy. Here, we report the generation of CD38-specific trimeric antibody recruiters (CD38-STARs) using nanobodies, single variable immunoglobulin domains isolated from camelid heavy-chain antibodies.

**Methods:**

We genetically fused two different CD38-specific nanobodies against distinct CD38 epitopes to a human immunoglobulin κ light chain-specific nanobody to generate two CD38-STARs (E2-E3-STAR and E3-E2-STAR) with biparatopic antigen-binding modules. We analyzed their CD38 binding characteristics and the capacity for simultaneous engagement of CD38 and IgG, IgM, or IgA. We evaluated the induction of complement-dependent cytotoxicity (CDC) against various CD38-expressing tumor cell lines *in vitro* and against primary MM patient cells *ex vivo*, comparing the CD38-STARs with monoparatopic bispecific antibody recruiters (BARs).

**Results:**

Both CD38-STARs engaged CD38 by simultaneously binding two epitopes that are distinct from the epitope bound by daratumumab, resulting in improved avidity compared to monoparatopic CD38-specific BARs. CD38-STARs demonstrated concurrent binding to CD38 and human IgG, IgM, or IgA. CD38-STARs induced higher CDC against various CD38-expressing tumor cell lines *in vitro* compared to monoparatopic BARs or daratumumab. *Ex vivo*, CD38-STARs mediated significant CDC induction against primary myeloma cells, whereas monoparatopic BARs failed to achieve significant killing.

**Conclusion:**

Biparatopic targeting of CD38 using CD38-STARs represents a promising strategy for the treatment of MM, including the rare cases in which daratumumab fails to bind due to CD38 mutations.

## Introduction

Multiple myeloma (MM) is a hematological disorder defined by unrestricted clonal proliferation of malignant plasma cells. With an age-standardized incidence rate of 1.79 per 100,000 persons, MM is one of the most frequent hematologic malignancies (1). To this day, MM remains an incurable disease, as most patients eventually become therapy-refractory and relapse (1–3). The associated mortality remains a significant global challenge, with the disease responsible for 116,000 deaths worldwide in 2021 (4). Projections indicate that the global incidence of MM will increase substantially in the coming years (5). Consequently, the development of novel therapeutic modalities to overcome these challenges remains a critical priority in MM research. Immunotherapeutic approaches in MM treatment have rapidly evolved based on increasingly complex antibody-based constructs including bispecific antibodies, T cell engagers, and chimeric antigen receptors (CARs) (6–10).

CD38 is an established target in MM therapy, as demonstrated by the clinical success of the CD38-specific monoclonal antibodies daratumumab and isatuximab (11, 12). However, eventual antigen loss or point mutations within the respective antibody-binding epitopes can lead to tumor escape and therapy resistance in patients treated with CD38-specific agents (13). Biparatopic targeting of CD38 offers a potential strategy to overcome point-mutation induced treatment resistance by mediating CD38 binding via two distinct epitopes (14). Additional key advantages of this approach include enhanced avidity and efficacy (14). The clinical impact of biparatopic targeting of MM cells has been demonstrated by the success of the nanobody-based CAR T cell therapy ciltacabtagene autoleucel (cilta-cel). By simultaneously engaging two distinct BCMA epitopes, this construct facilitated unprecedented response rates in MM patients (10). Nanobodies are single variable immunoglobulin domains derived from camelid heavy-chain antibodies (**Figure 1A**) (15, 16). They demonstrate high solubility (16), low immunogenicity (16), and high ease of reformatting (17), making them ideally suited as building blocks in recombinant antibody constructs.

**Figure 1:**
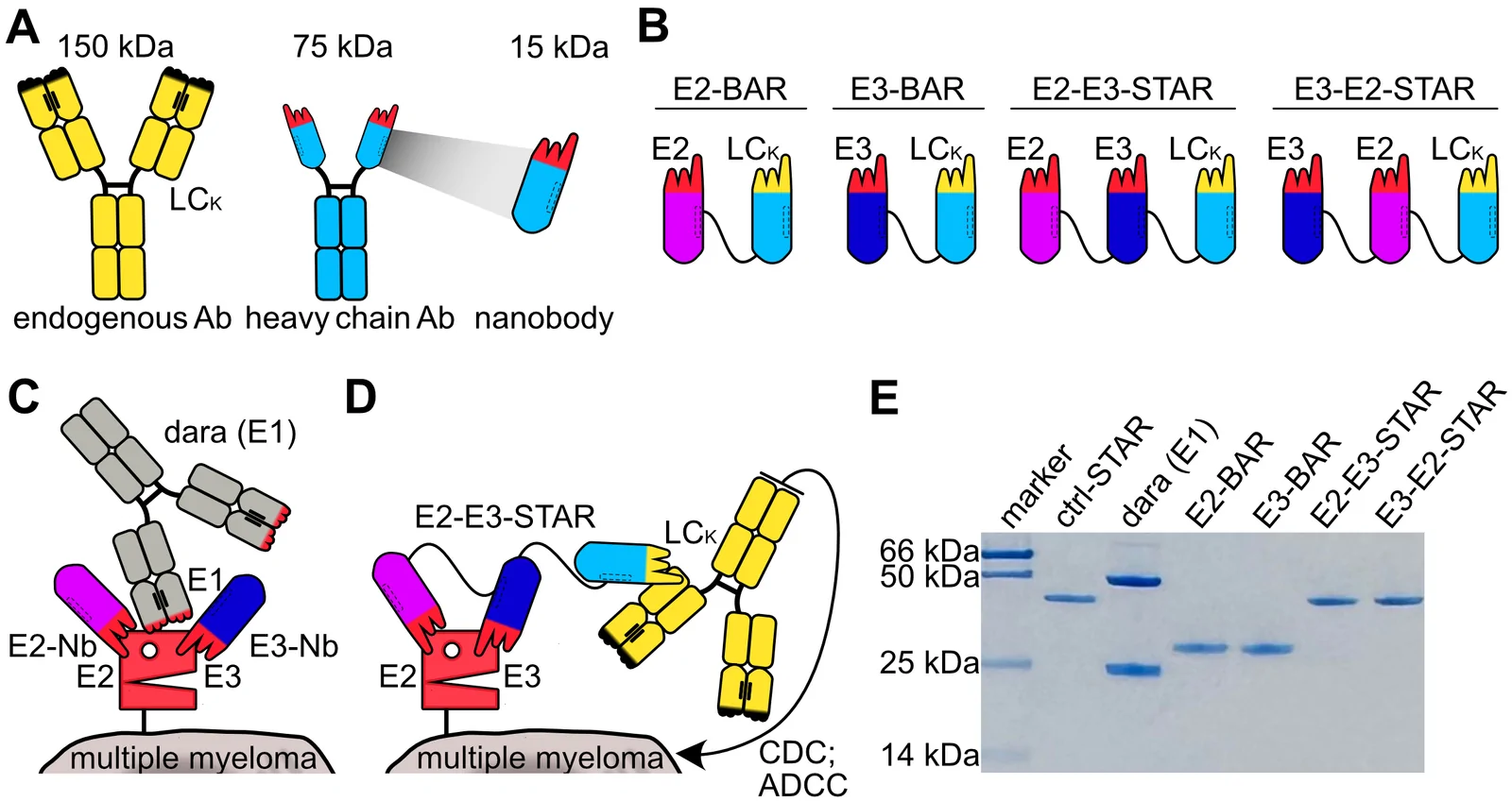
Schematics and characterization of conventional antibodies, heavy chain antibodies, nanobodies, bispecific antibodies (BARs), and CD38-specific trimeric antibody recruiters (CD38-STARs). **(A)** The structure of endogenous antibodies (Abs) is formed by two heavy and two light chains. Their antigen-binding site is composed of two hydrophobically associated variable domains. In comparison, heavy chain antibodies (hcAbs) consist of two heavy chains and lack the light chains. Their antigen-binding site is formed by a single variable domain, which, when produced recombinantly, is called a nanobody (Nb). Nbs exhibit high solubility due to the replacement of the hydrophobic interface with a hydrophilic region (dashed lines). **(B)** Bispecific antibody recruiters (BARs) consist of two Nbs fused via a flexible glycine-serine linker: one Nb specific for CD38 (E2-Nb, purple; E3-Nb, blue) and one Nb specific for the kappa light chain of endogenous Abs (LC_k_-Nb, turquoise). CD38-specific trimeric antibody recruiters (CD38-STARs) represent an extension of the CD38-specific BARs. This design includes an additional Nb that targets a distinct epitope on CD38, allowing biparatopic binding to CD38. **(C)** E2-Nb (targeting epitope 2; purple) and E3-Nb (targeting epitope 3; blue) bind to distinct regions on CD38 expressed by multiple myeloma cells. Binding of the STARs does not interfere with binding of the clinical approved antibody daratumumab (dara, grey) targeting epitope 1 (E1), thus permitting simultaneous use. **(D)** E2-E3-STAR binds biparatopically to CD38 while simultaneously recruiting circulating endogenous Abs via its kappa light chain-specific Nb. Recruited immunoglobulins then induce Fc-dependent effector functions such as CDC and ADCC. **(E)** SDS-PAGE analysis of purified ctrl-STAR, dara, CD38-specific BARs, and CD38-STARs. Proteins (1 µg per lane) were size fractionated under reducing conditions and stained with Coomassie Blue.

We previously demonstrated the principal therapeutic potential of CD38-specific nanobody-based bispecific antibody recruiters (BARs) for the treatment of MM (18). These BARs consist of an immunoglobulin κ light chain-specific nanobody linked via a flexible glycine-serine linker to a monoparatopic CD38-specific nanobody. This enabled the recruitment of circulating antibodies to the MM cell surface, thereby initiating Fc-dependent effector functions such as complement-dependent cytotoxicity (CDC) and antibody-dependent cellular cytotoxicity (ADCC).

We hypothesized that incorporation of an additional nanobody specific for a distinct epitope of CD38 into CD38-specific BARs would result in biparatopic tumor binding conferring improved therapeutic efficacy. Additionally, this approach represents a potential strategy to overcome treatment resistance mediated by epitope loss during CD38-targeted therapy.

The aim of this study was to engineer CD38-specific trimeric antibody recruiters (CD38-STARs) that bind biparatopically to CD38 and to evaluate their cytotoxic potential against myeloma cells *in vitro* and *ex vivo* compared to monoparatopic CD38-specific BARs.

## Materials and methods

### Cell lines

HEK293-6E cells were used under license of Yves Durocher, Ottawa, Canada. The cell lines LP-1 (human MM), Daudi and CA-46 (human Burkitt lymphoma), NK92, Chinese hamster ovary cells (CHO), and HEK293T cells were obtained from the German Collection of Microorganisms and Cell Culture (DSMZ, Braunschweig, Germany). LP-1 luc, Daudi luc, and CA-46 luc cell lines stably expressing GFP and the luc2 variant of *Photinus pyralis* luciferase (Promega, Madison, WI, USA) under the control of the spleen-focus-forming virus U3 region (SFFV promoter) were generated by lentiviral transduction as described previously (19–21). As previously reported (22), tumor cells transduced with GFP and luciferase were selected using puromycin and maintained in culture. GFP and luciferase expression were monitored regularly via flow cytometry and luciferase-based bioluminescence assays. Surface expression of CD38 was assessed via flow cytometry by staining cells with the CD38-targeting, Alexa Fluor 647 (AF647)-conjugated heavy chain antibody JK2 (23) at a 1:500 dilution for 20 minutes at 4 °C.

NK92 cells were engineered to stably express high levels of human CD16 and eGFP via retroviral transduction using the pSF91 retroviral vector (24). The human CD16 sequence, i.e., the ectodomain of FcγRIII fused to the transmembrane and cytosolic domains of FcϵRI, was obtained from Béatrice Clémenceau (Nantes, France) (25). To prevent CD38-mediated fratricide, the CD38 gene was knocked out using CRISPR/Cas9 (cat. #sc-401117-NIC, Santa Cruz Biotechnology, Dallas, TX, USA), as previously described (26). Cells were maintained in alpha-MEM culture medium (Gibco) containing 10% horse serum (Gibco), 10% fetal bovine serum (Gibco), 5 mM L-glutamine (Gibco), and 5 ng/mL IL-2 (Proleukin, Novartis, Nürnberg, Germany).

To generate HEK293T cells stably expressing human CD38, we used polyethylenimine-mediated transfection with cDNA expression vectors additionally coding for a puromycin resistance. Transfected cells were isolated via fluorescence-activated cell sorting and cultivated in DMEM culture medium (Gibco) containing 4.5 g/L glucose, 1 µg/mL puromycin, 5% (v/v) fetal bovine serum (Gibco), 2 mM sodium pyruvate (Gibco), and 2 mM L-glutamine (Gibco).

HEK293T cells and CHO cells transiently expressing the mutations CD38^S274F^ or CD38^K71I^ were generated via polyethylenimine-mediated transfection with pCSE2.5 expression vectors kindly provided by Thomas Schirrmann, Braunschweig, Germany). CD38^S274F^ is a specifically characterized mutation known to lead to the loss of daratumumab binding (27). The CD38^K71I^ mutation was artificially introduced at the presumed binding epitope of the CD38-specific nanobody JK2 (E2). It was assumed to alter binding of JK2 based on protein structure predictions generated by AlphaFold (Google DeepMind, Mountain View, CA, USA). Both mutant constructs were generated based on the same CD38 nucleotide sequence. Cells were used for experiments 2 days after transfection.

To stably express CD38^L153H^, a clinical resistance mutation recently identified in a single myeloma patient relapsing after anti-CD38 therapy (13), we utilized LP-1 luc cells in which CD38 was previously knocked out as described above. These cells were transduced with lentiviral vectors encoding either the CD38^L153H^ mutant or wild-type CD38 (CD38^wt^), alongside a blasticidin resistance gene. Transduced cells were routinely monitored for CD38 expression and cultured in RPMI medium (Gibco) supplemented with 10 µg/mL blasticidin, 5% (v/v) fetal bovine serum (Gibco), 2 mM sodium pyruvate (Gibco), and 2 mM L-glutamine (Gibco).

### Antibody recruiters and conventional antibodies

Generation of the human CD38-specific nanobodies JK2, JK36, and the control nanobody L-10e was performed in immunized llamas as described previously (28, 29).

CD38-specific BARs were generated as described previously (18). Briefly, CD38-specific nanobodies JK2 and JK36 (23) were genetically fused to a nanobody specific for the human κ light chain (Hu-kappa-1), obtained from patent WO 2006/059904 (30). Nanobody L-10e targeting the cysteine protease domain of *Clostridioides difficile* toxin was used as an isotype control (31). To generate trimeric STAR constructs, two CD38-specific nanobodies (or L-10e) were fused via a 15-amino-acid glycine-serine (GS) linker using overlap extension PCR. The resulting PCR products were subcloned into the pCSE2.5 vector upstream of Hu-kappa-1, connected by an additional flexible 15-amino-acid GS linker.

For simplified nomenclature, the generated BAR and STAR constructs containing the CD38-specific nanobodies JK2 (binding epitope E2) and JK36 (binding epitope E3) fused to Hu-kappa-1 are designated as E2-BAR, E3-BAR, E2-E3-STAR, and E3-E2-STAR. The fusion of two L-10e domains to Hu-kappa-1 is designated as ctrl-STAR.

The previously published dissociation rates (k_diss_) for these nanobodies are 1.1×10^−3^s^−1^ (JK2) (23), 2.0×10^−4^s^−1^ (JK36) (23), and 7.8×10^−4^s^−1^ (Hu-κ-1) (30), whereas a corresponding value for L-10e has not yet been determined. We anticipate that the kinetic properties of the individual nanobodies are conserved in the BAR and STAR constructs, given that the flexible glycine-serine linker facilitates autonomous domain folding and binding (32).

BARs and STARs were produced in transiently transfected HEK293-6E cells cultivated in serum-free medium. Supernatants were harvested six days post transfection and clarified by centrifugation. BARs and STARs were purified by affinity chromatography using Protein A sepharose (33). The purity of the constructs was verified via SDS-PAGE and InstantBlue Coomassie staining.

Daratumumab (Darzalex, Janssen-Cilag, Neuss, Germany) was obtained to serve as the positive control in the killing assays.

### Binding analyses

Simultaneous binding of CD38-specific BARs and CD38-STARs to CD38 and human IgGκ, IgMκ, IgAκ was analyzed using HEK293T cells stably transfected with human CD38. Cells were incubated with 100 nM of the CD38-STARs, CD38-specific BARs, ctrl-STAR, or buffer alone (PBS + 0.2% BSA) for 20 min at 4 °C. Daratumumab (100 nM) served as a positive control for IgGκ detection and as a negative control for IgMκ and IgAκ staining. Human pooled serum (25%) was added to provide polyclonal human immunoglobulins, followed by 20 min incubation at 4 °C. After washing, bound IgG, IgA, and IgM were stained using PE-conjugated, human IgG-specific donkey IgG F(ab’)2 (cat. #709-116-098), human IgA-specific goat IgG (cat. #109-115-011), and human IgM-specific donkey IgG F(ab’)2 (cat. #709-116-073) detection antibodies (all from Jackson ImmunoResearch, West Grove, PA, USA). Following two final wash steps, samples were measured by flow cytometry using a FACSymphony A1 (Becton Dickinson, Franklin Lakes, NJ, USA) and analyzed using FlowJo v10 software (Becton Dickinson).

To evaluate potential biparatopic binding of CD38-STARs and assess competition with daratumumab, blocking assays were performed on LP-1 luc MM cells using STARs, BARs, and daratumumab conjugated to AF647 (Invitrogen, Karlsbad, CA, USA). Labeling was performed according to the manufacturer’s instructions. Cells were pre-incubated for 20 min with either buffer (PBS + 0.2% BSA), 100 nM single-chain variable fragment (scFv) of daratumumab (dara-scFv), 100 nM E2-Nb, 100 nM E3-Nb, or a combination of E2-Nb and E3-Nb (100 nM each) to saturate CD38 epitopes. Dara-scFv was used to prevent false-positive signals resulting from the binding of κ-specific nanobodies to daratumumab’s light chain. This was followed by the addition of AF647-labeled constructs at a 1:250 dilution (v/v) and incubation for an additional 20 min at 4 °C. Following two washing steps, samples were measured by flow cytometry (FACSymphony A1) and analyzed using FlowJo v10. The percentage decrease in AF647 median fluorescent intensity (MFI) on blocked cells compared to unblocked cells was calculated using GraphPad Prism 10.2.3 (GraphPad, Boston, MA, USA).

Differences in avidity to CD38 were assessed by measuring the relative dissociation rates of the constructs from the surface of LP-1 luc cells. Cells were stained with saturating amounts (1:250) of fluorochrome-conjugated BARs, STARs, or daratumumab for 20 min at 4 °C. After washing three times, the loss of cell-associated fluorescence was monitored over time at room temperature. To serve as a sink for dissociated antibodies, LP-1 luc cells labeled with the cell-tracking dye eFluor 450 (Invitrogen) were added to the samples. Cell-associated fluorescence was measured immediately upon addition of sink cells (*t* = 0). Dissociation was then monitored by measuring fluorescence at defined time points (5, 10, 15, 30, 45, and 60 min) following the addition of the eFluor-labeled sink cells. Data was acquired by flow cytometry (FACSymphony A1) and analyzed using FlowJo v10. MFI changes were calculated relative to the baseline value (*t* = 0) of the respective construct and visualized using GraphPad Prism 10.2.3.

Binding to HEK293T cells expressing mutated CD38 variants was assessed by incubation with AF647-labelled BAR and STAR constructs for 20 min at 4 °C. Transfected cells were detected via staining with a His-tag-specific, FITC-labelled antibody (cat. #A01620, GenScript, Nanjing, China). Following two washing steps, samples were measured by flow cytometry (FACSymphony A1) and analyzed using FlowJo v10.

### CDC of tumor cell lines

Daudi luc, CA-46 luc, LP-1 luc, LP-1 luc CD38^wt^, and LP-1 luc CD38^L153H^ cells were treated with 100 nM or serial dilutions of E2-BAR, E3-BAR, E2-E3-STAR, E3-E2-STAR, ctrl-STAR, or daratumumab in 100 µL PBS containing 0.2% BSA for 20 min at 37 °C. To provide complement and unspecific, polyclonal antibodies, samples were supplemented with 25% human pooled serum and incubated for an additional 90 min at 37 °C. Heat-inactivated serum (30 min at 56 °C) served as a negative control. After a single washing step, cells were resuspended in PBS containing 0.2% BSA and 37.5 µg/mL D-luciferin (Biosynth, Staad, Switzerland). After 25 min incubation at 37 °C, bioluminescence was recorded using a microplate reader (Victor^3^, PerkinElmer, Waltham, MA, USA). The percentage of CDC induction was calculated as follows:


$$\%\text{live cells}=\frac{\text{BLI signal }\;[\text{sample}]}{\text{BLI signal }\;[\text{ctrl}‐\text{STAR}]}\;\times \;100\%$$

To evaluate combinatorial effects, LP-1 luc cells were treated with 100 nM daratumumab or with 100 nM daratumumab + EC_50_ of STARs/BARs. 100 nM daratumumab + 100 nM ctrl-STAR was used as a negative control. The CDC assay was performed as described above.

CDC assays involving epitope pre-blockade were performed using LP-1 luc cells. Cells were pre-incubated for 20 min at 37 °C with 200 nM dara-scFv, 200 nM E2-Nb, or 200 nM E3-Nb. Following pre-incubation, the CDC assay was carried out as described above.

For CDC kinetics, LP-1 luc cells were first treated with STARs, BARs, or control constructs as described above. To establish a stable luminescence baseline signal, cells were incubated with 50 µg/mL D-luciferin for 20 min at 37 °C before adding 25% human pooled serum. Luminescence kinetics were monitored at 1-minute intervals for a duration of 90 min, starting 180 seconds after the addition of serum.

To assess cytotoxicity against mutated CD38 targets, CHO cells transiently expressing the CD38 mutations CD38^S274F^ or CD38^K71I^ were used in a CDC assay as described before and quantified via flow cytometry (FACSymphony A1). Gating was performed on live cells and transfected target cells were identified using the previously mentioned His-tag-specific antibody. Target cell survival was evaluated by determining the percentage of His-positive (transfected) cells relative to the His-negative (non-transfected) cell population within the same sample. To quantify specific cytotoxicity, the viability of target cells treated with the therapeutic constructs was normalized to the ctrl-STAR, which was set to 100%:


$$\%\text{live cells}=\frac{\%\text{His-positive cells }\;[\text{sample}]}{\%\text{His-positive cells }\;[\text{ctrl}‐\text{STAR}]}\times 100\%$$

Data were analyzed using FlowJo v10 and GraphPad Prism 10.2.3.

### Protein structure visualization

Structural visualizations of the human CD38 extracellular domain were generated using UCSF ChimeraX (version 1.12, San Francisco, CA, USA). The crystal structure PDB 4CMH served as the structural template. The daratumumab binding epitope (E1) was defined as amino acid regions 233–246 and 267–280, based on established peptide mapping data (34). The isatuximab binding epitope (E2) was defined as all CD38 protein residues in direct contact with the isatuximab Fab chains B and C (35). The position of the clinical resistance mutation L153H (13) was highlighted on the protein structure.

### ADCC of tumor cell lines

Target cells were incubated in alpha-MEM with or without 100 nM of BARs, STARs, or daratumumab for 20 min at 37 °C before adding 25% heat-inactivated human pooled serum as a source of non-specific antibodies. Cells were incubated for 20 min at 37 °C followed by the addition of NK92 hCD16 CD38KO effector cells at an effector-to-target (E:T) ratio of 3:1. After 180 min incubation at 37 °C, cells were washed and resuspended in PBS supplemented with 0.2% BSA and 37.5 µg/mL D-luciferin. Bioluminescence was quantified after 20 min incubation at 37 °C, and viability was calculated relative to the ctrl-STAR as described above.

### CDC of primary myeloma cells

All experiments involving human participants were approved by the ethics committee of the Ärztekammer Hamburg (PV5505). Research was conducted in accordance with the Declaration of Helsinki, and written informed consent was obtained from all patients.

Primary myeloma cells were derived from myeloma patient bone marrow aspirates. Following the isolation of bone marrow mononuclear cells (BM-MNCs) via Ficoll-Paque density gradient centrifugation (Sigma-Aldrich, St. Louis, MO, USA), erythrocytes were depleted using red blood cell lysis buffer (NH_4_Cl + KHCO_3_ + EDTA). BM-MNCs were characterized by flow cytometry with a panel of fluorochrome-conjugated antibodies before further experiments: CD19 (clone HIB19, BD, cat. #555414), CD38 (clone JK2 (23)), CD55 (clone IA10, BD, cat. #555694), CD56 (clone B159, BD, cat. #560916), CD59 (clone p282, BD, cat. #555764), CD138 (clone MI15, BioLegend, cat. #356516), CD229 (clone hLy9.1.25, BioLegend, cat. #326108), CD269 (clone 19F2, BioLegend, cat. #357508), and CD319 (clone 162.1, BioLegend, cat. #331816). Cell viability was determined using the amine-reactive dye Pacific Orange succinimidyl ester (cat. #P30253, Thermo Fisher, Waltham, MA, USA) (36). MM cells were defined by their high expression of CD38 and CD138.

To evaluate CDC activity, BM-MNCs were treated with 100 nM BAR, STAR, or daratumumab in PBS supplemented with 0.2% BSA. To provide a source of complement and non-specific antibodies, 25% human pooled serum was added and cells were incubated for 90 min at 37 °C. Cells were washed once and then stained for flow cytometry using fluorochrome-conjugated antibodies specific for CD38, CD138, and CD229. Myeloma cells were subsequently characterized based on high expression levels of CD138 in combination with either CD38 or CD229. To avoid interference, CD38 was detected using AF647-conjugated nanobodies (clones WF211, MU1067, or JK36) that bound to an epitope distinct from that of the therapeutic constructs. Absolute cell numbers were quantified using CountBright counting beads (Invitrogen, Waltham, MA, USA). Data was processed in FlowJo v10. CDC induction was calculated as follows:


$$\%\text{live cells}=\frac{\text{MM cell count per µL}\;[\text{sample}]}{\text{MM cell count per µL}\;[\text{ctrl}‐\text{STAR}]}\times 100\%$$

### Statistical analysis

Statistical analysis was performed using GraphPad Prism 10.2.3, with results expressed as mean ± standard deviation (SD). Assuming a normal distribution of the *in vitro* data, inter-group differences were assessed via one-way ANOVA. Dunnett’s multiple comparisons test was used to analyze differences between the ctrl-STAR and therapeutic constructs, while Tukey’s multiple comparisons test was applied for pairwise comparisons between the therapeutic constructs (CD38-STARs, BARs, and daratumumab). EC_50_ values for *in vitro* CDC were calculated via non-linear regression using a four-parameter logistic (4PL) model on data normalized to the ctrl-STAR. For *ex vivo* analyses, differences between groups were evaluated using the non-parametric Friedman test. Dunn’s multiple comparisons test was applied to compare treatments with the ctrl-STAR and across therapeutic pairs. A *p* value < 0.05 was considered statistically significant for all tests.

## Results

### Biparatopic STARs enable stronger binding to CD38 than monoparatopic BARs or daratumumab

To generate biparatopic CD38-STARs, two nanobodies targeting distinct epitopes on CD38 (designated E2 and E3) were genetically fused to a nanobody specific for the human immunoglobulin κ light chain (**Figures 1B–D**). These trimeric constructs were generated in two variants (E2-E3-LCκ and E3-E2-LCκ) to assess whether the orientation of the CD38-targeting nanobodies relative to each other would affect CD38 binding (**Figure 1B**). CD38-STARs were purified from the supernatants of transiently transfected HEK293-6E cells, and their structural integrity and purity were confirmed by SDS-PAGE (**Figure 1E**). Monoparatopic CD38-specific BARs targeting single epitopes (E2-LCκ and E3-LCκ) and an isotype control-STAR (ctrl-STAR) targeting an irrelevant antigen served as controls.

Simultaneous binding of the CD38-STARs to both CD38 and human immunoglobulin κ light chain was confirmed by flow cytometry (**Figure 2**). CD38-transfected HEK293T cells were incubated with CD38-specific BARs, CD38-STARs, or a ctrl-STAR in the presence of 25% heat-inactivated human serum as a source of polyclonal immunoglobulins. Staining with PE-conjugated hIgG-, hIgM-, and hIgA-specific secondary antibodies (**Figures 2A–C**) revealed simultaneous binding to CD38 and to serum IgG, IgM, and IgA for both CD38-specific BARs and CD38-STARs. In contrast, no signal was observed for the ctrl-STAR or for cells incubated without an antibody recruiter. Daratumumab was used to confirm cell surface expression of CD38. Daratumumab (IgG1) also served as positive and negative controls for the isotype-specific binding of the secondary anti-human IgG, IgA, and IgM antibodies.

**Figure 2:**
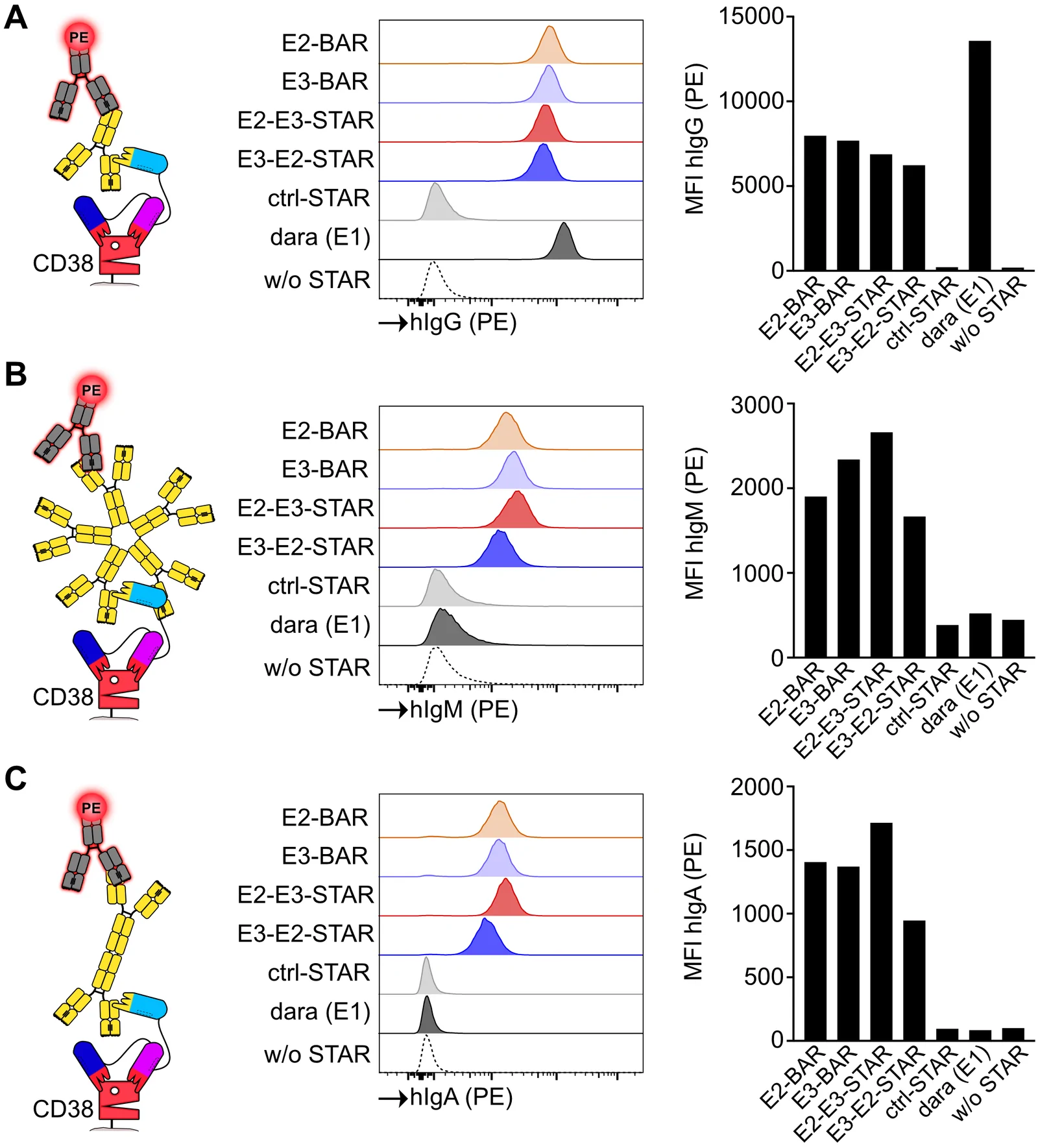
CD38-STARs specifically and simultaneously bind CD38 and the human kappa light chain of IgM, IgA, and IgG. **(A)** Schematic illustration of the experimental setup to verify binding of antibody recruiters to both CD38 and the kappa light chain of human antibodies. CD38-specific BARs and CD38-STARs were incubated with CD38-transfected HEK cells for 20 min. Human pooled serum was added as a source of IgGκ (yellow). A PE-conjugated antibody specific for human IgG (grey) was added to detect STAR-mediated binding of human antibodies to CD38. Flow cytometry histograms demonstrate recruitment of IgGκ to CD38 via BARs and STARs (colored histograms). Daratumumab (dara, black histogram) served as a control for the isotype specificity of the secondary antibodies. Samples incubated with an unspecific ctrl-STAR (grey histogram) or without STAR (dashed histogram) were used as negative controls. Bars represent median fluorescence intensities (MFI). **(B)** Experimental setup, flow cytometry histograms, and quantification of BAR and STAR binding to IgMκ using a hIgM-specific secondary antibody. Dara served as a negative control (black histogram). **(C)** Experimental setup, flow cytometry histograms, and quantification of BAR and STAR binding to IgAκ using a hIgA-specific secondary antibody. Dara served as a negative control (black histogram).

To evaluate biparatopic binding of CD38-STARs and potential competition with daratumumab, we performed competitive binding analyses (**Figure 3**). LP-1 luc cells were pre-incubated with nanobodies specific for epitope 2 or 3 of CD38 (individually or in combination) or with dara-scFv, a single chain antibody derived from daratumumab, to block epitope 1. This was followed by incubation with AF647-labeled BARs, STARs, or dara-scFv. The latter was used instead of daratumumab to prevent binding of the antibody recruiters to daratumumab’s κ light chain. Pre-blocking of their respective target epitope strongly reduced binding of both CD38-specific BARs (**Figures 3A, B**). In contrast, binding of CD38-STARs was unaffected or only moderately reduced by blockade of one of their two targeted epitopes but strongly reduced by simultaneous blockade of both of them. These results indicate a biparatopic binding mechanism of CD38-STARs.

**Figure 3:**
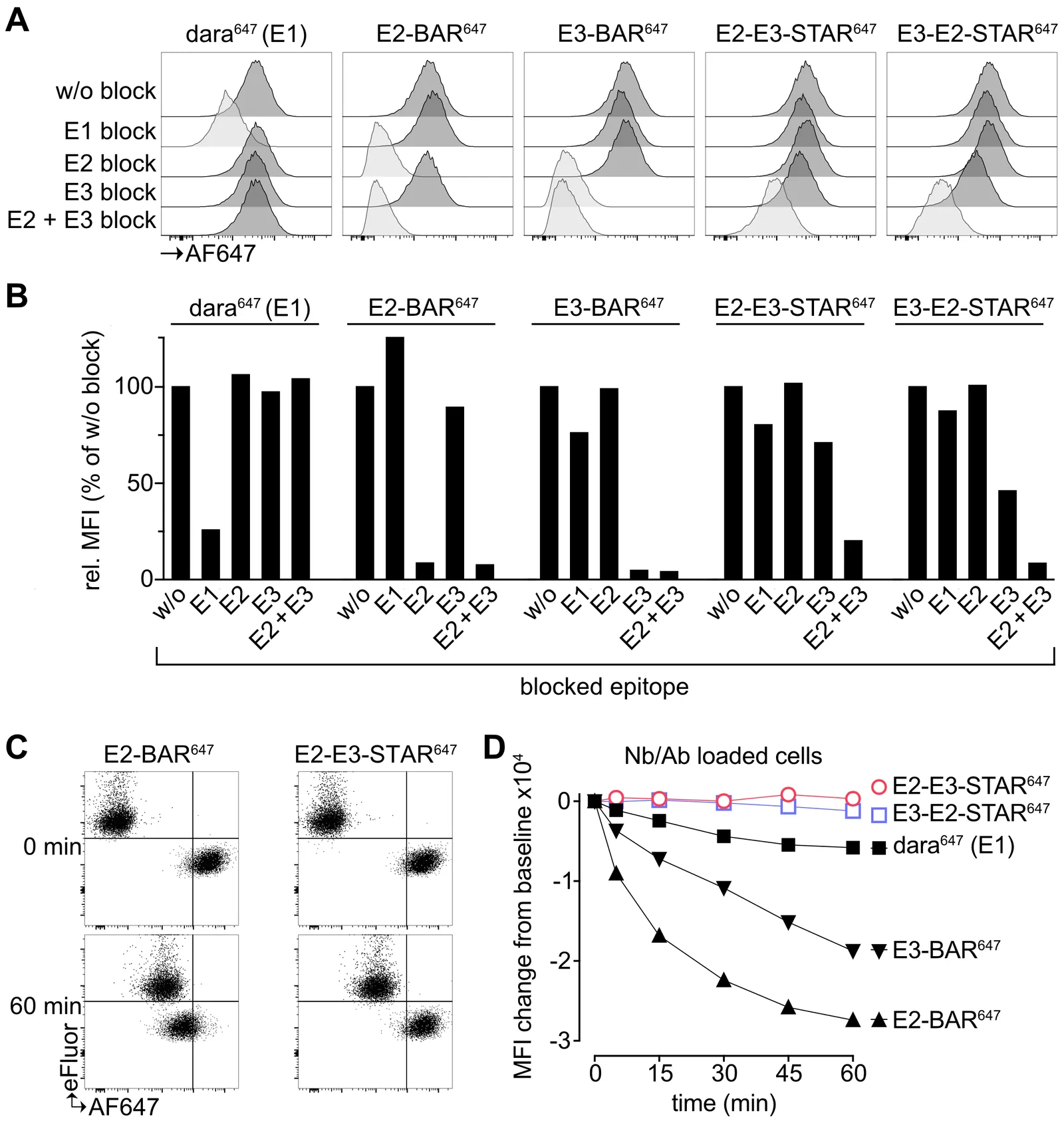
CD38-STARs show enhanced binding to CD38 compared to CD38-specific BARs and daratumumab. **(A)** Flow cytometry histograms demonstrating competitive binding of Alexa Fluor 647-labeled daratumumab (dara^647^), CD38-specific BARs, and CD38-STARs to LP-1 luc cells. Binding was assessed without blocking (w/o block) or after pre-incubation with dara-scFv (E1 block), E2-Nb (E2 block), E3-Nb (E3 block), or a combination of E2-Nb and E3-Nb (E2 + E3 block). **(B)** Quantification of binding shown in **(A)**. Bars represent the relative median fluorescence intensity (MFI) expressed as a percentage of the respective unblocked construct (set to 100%). **(C)** Representative dot plots and **(D)** dissociation kinetics of fluorochrome-conjugated antibody recruiters and daratumumab (dara^647^) bound to LP-1 luc cells, indicated by MFI changes. Cells were first incubated for 20 min with 100 nM of AF647-conjugated constructs (Nb/Ab loaded cells). Cells were then washed three times. LP-1 luc sink cells pre-labeled with eFluor 450 were added at *t* = 0 and the loss of cell-associated fluorescence was monitored over time.

To assess the avidities of our BARs and STARs, we next analyzed their relative rates of dissociation from LP-1 cells (**Figures 3C, D**). An aliquot of LP-1 cells was incubated with saturating amounts of AF647-conjugated antibody recruiters or daratumumab. After removal of unbound antibodies by washing, we added a second aliquot of unstained LP-1 cells and observed migration of the AF647-conjugates from the pre-stained to the unstained cell population over 60 min. The results demonstrate slower dissociation of CD38-STARs compared to CD38-specific BARs and daratumumab, consistent with superior avidity of the CD38-STARs.

### STARs mediate stronger CDC *in vitro* than BARs or daratumumab

The complement-dependent cytotoxicity (CDC) induced by CD38-STARs was assessed against three different luciferase-expressing tumor cell lines (Daudi, CA-46, and LP-1) using human serum as a source of complement (**Figure 4**). These cell lines express high and uniform cell surface levels of CD38 (**Figure 4A**).

**Figure 4:**
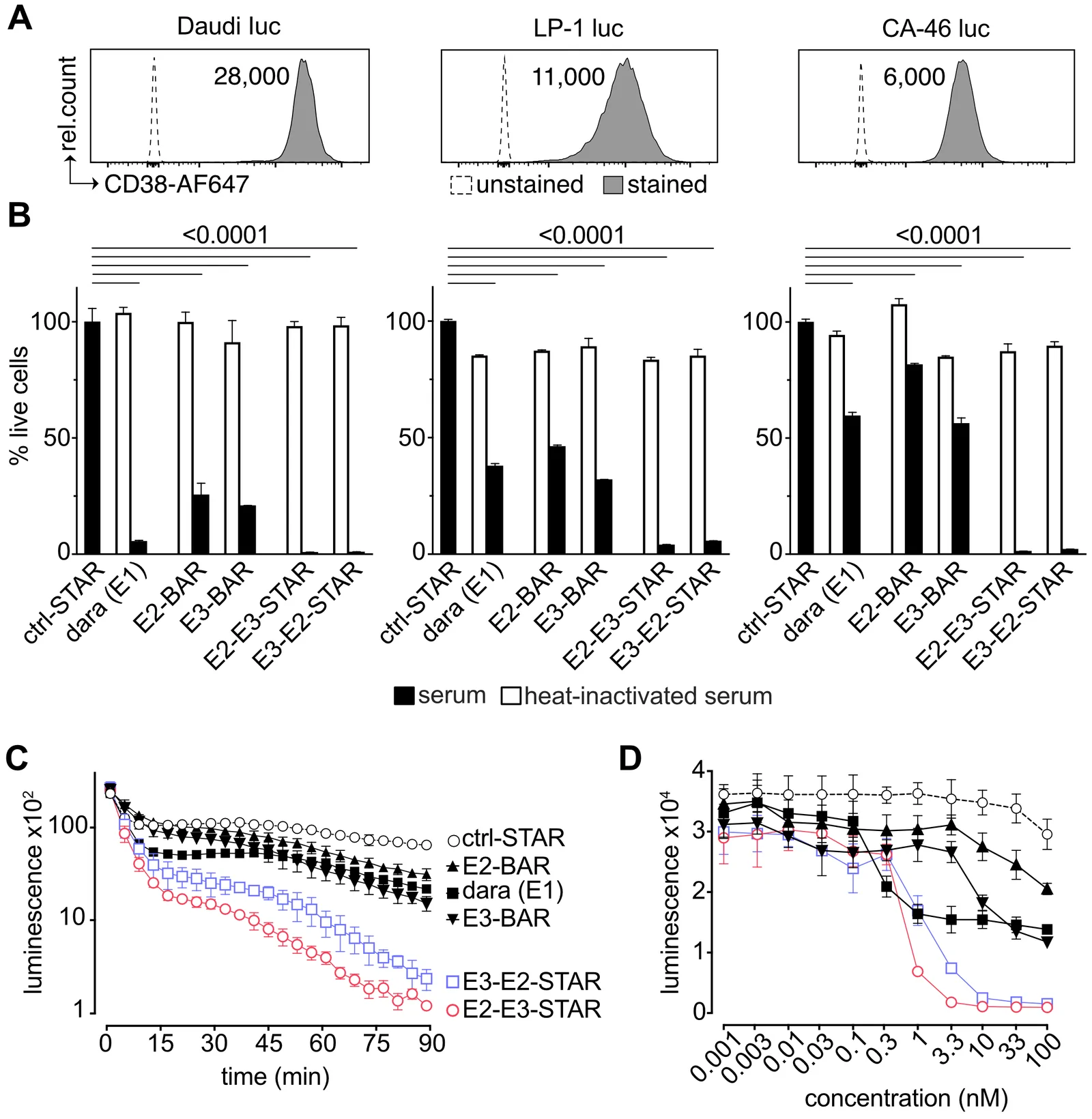
CD38-STARs mediate time- and dose-dependent complement-dependent cytotoxicity (CDC) against CD38-expressing cell lines *in vitro*. **(A)** CD38 cell surface levels on Daudi luc, LP-1 luc, and CA-46 luc cells were analyzed by staining with Alexa Fluor 647 (AF647)-conjugated heavy chain antibody JK2. The corresponding MFI of the stained cells is shown next to each histogram. **(B)** Viability of Daudi luc, LP-1 luc, and CA-46 luc cells was evaluated following 90 min of incubation with 100 nM of CD38-specific BARs, CD38-STARs, ctrl-STAR, or daratumumab (dara) in the presence of 25% human serum as a source of immunoglobulin and complement (black bars). Heat-inactivated serum served as a negative control (white bars). Bioluminescence was quantified as an indicator of cell viability. Cells incubated with ctrl-STAR were set as 100% viable cells. Results of a one-way ANOVA with Dunnett’s multiple comparisons test revealed significant differences between ctrl-STAR and all samples after incubation with serum (*p* < 0.0001). **(C)** Time-resolved kinetics of LP-1 cell viability over 90 min. Cells were treated with 100 nM of the indicated STAR constructs, and 25% human pooled serum was added at *t* = 0. Luminescence was measured every minute. **(D)** Dose dependence of CDC against LP-1 cells was determined after incubation with the indicated concentrations of STARs, BARs, and dara in the presence of 25% human pooled serum for 90 min. Data represent mean ± SD.

CD38-STARs, CD38-specific BARs, and daratumumab induced significant killing of all cell lines within 90 min compared to the ctrl-STAR (**Figure 4B**). Cytotoxicity was complement-dependent, as confirmed by lack of killing in samples incubated with heat-inactivated serum. Both CD38-STARs exhibited higher cytotoxicity compared to CD38-specific BARs. This effect was statistically significant across all cell lines for every pairwise comparison (Daudi luc *p* < 0.01; LP-1 luc and CA-46 luc *p* < 0.0001). Additionally, both CD38-STARs showed significantly increased killing compared to daratumumab in LP-1 luc and CA-46 luc cells (both *p* < 0.0001), but not in Daudi luc cells (both *p* > 0.05). CDC efficacy of the CD38-STARs was uniform and strong, achieving >95% lysis in all cell lines independent of CD38 cell surface levels. By contrast, CDC efficacy of both daratumumab and monoparatopic CD38-specific BARs decreased with decreasing levels of cell surface CD38. E2-E3-STAR induced marginally higher CDC activity than E3-E2-STAR in all cell lines, although this difference was not statistically significant (all *p* > 0.05).

CD38-STARs demonstrated superior CDC efficacy in kinetic and dose-response assays against LP-1 luc cells compared to daratumumab or CD38-specific BARs (**Figures 4C, D**). Nonlinear regression analysis was performed on data normalized to the negative control (ctrl-STAR, 0% lysis) to determine the intrinsic potency (EC_50_) and efficacy (E_max_) of the constructs. E2-E3-STAR showed the strongest potency and efficacy (EC_50_: 0.65 nM, E_max_: 96.6%), followed by E3-E2-STAR (EC_50_: 1.3 nM, E_max_: 96.1%). In comparison, E3-BAR showed only moderate potency and efficacy (EC_50_: 7.6 nM, E_max_: 60.5%). E2-BAR showed the least potency and efficacy (EC_50_ and E_max_ unstable). Due to the absence of a distinct plateau at the highest concentration of 100 nM, the four-parameter logistic (4PL) fit for the log (EC_50_) was unstable. To obtain a quantifiable potency estimate, the Hill slope was constrained to 1.0. Under this restriction, E2-BAR showed an EC_50_ of 13.27 nM and an E_max_ of 33.4%.

### CD38-STARs synergistically enhance daratumumab efficacy and retain cytotoxicity in the presence of single-epitope blockade and CD38 mutations

Possible interference between daratumumab and STARs during CDC induction was assessed in a CDC assay combining 100 nM daratumumab with BARs or STARs (**Figure 5A**). BARs and STARs were added at their median effective concentrations (EC_50_) to uncover potential synergistic effects and to account for their high baseline killing capacity. The results show that combining 100 nM daratumumab with the EC_50_ of E2-BAR, E3-BAR, E2-E3-STAR, or E3-E2-STAR resulted in a significant increase in killing compared to daratumumab alone (all *p* < 0.0001). Compared to the respective construct used individually, the combined treatment with daratumumab achieved a significant enhancement in CDC across all CD38-specific BARs and CD38-STARs (all *p* < 0.0001). Adding 100 nM of ctrl-STAR did not impair daratumumab-mediated killing.

**Figure 5:**
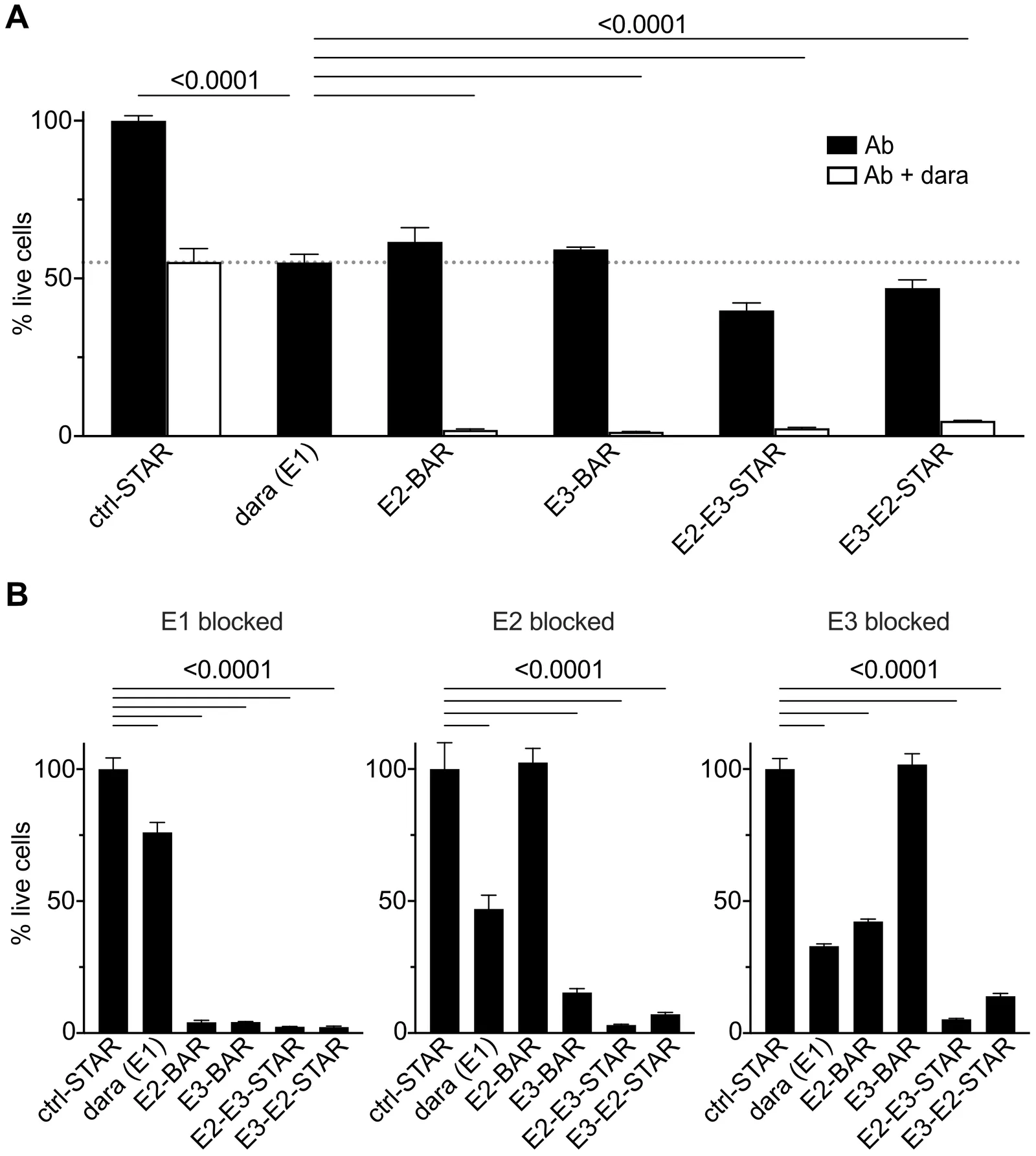
CD38-STARs demonstrate synergistic killing with daratumumab and retain cytotoxicity after E1, E2, or E3 epitope blockade. **(A)** Synergistic effects of CD38-specific BARs and CD38-STARs in combination with daratumumab (dara) on complement-dependent cytotoxicity (CDC) against LP-1 luc cells. Black bars represent single-agent treatments with 100 nM ctrl-STAR, dara, or EC_50_ of BARs and STARs. White bars indicate the combination of the constructs (at EC_50_) with 100 nM dara. The dotted grey line indicates killing achieved by 100 nM dara alone. Data were normalized to the ctrl-STAR (set to 100%). Statistical significance compared to 100 nM dara was determined using a one-way ANOVA followed by Dunnett’s multiple comparisons test and *p*-values are indicated. **(B)** CDC after epitope blocking on LP-1 luc cells. Cells were pre-incubated with dara-scFv (E1 blocked), E2-Nb (E2 blocked), or E3-Nb (E3 blocked) prior to CDC killing assays. Data were normalized to the ctrl-STAR (set to 100%). Statistical significance between the respective samples and the ctrl-STAR was determined using a one-way ANOVA followed by Dunnett’s multiple comparisons test and *p*-values are indicated.

To investigate whether CD38-STARs mediate CDC independently of the epitope targeted by daratumumab (E1) and to evaluate target engagement after individual binding sites had been compromised, LP-1 luc cells were incubated with dara-scFv, E2-Nb, or E3-Nb prior to the CDC assay (**Figure 5B**). Following pre-blockade with dara-scFv, CDC by daratumumab was markedly reduced, whereas CD38-specific BARs and CD38-STARs maintained robust cytotoxicity. Pre-blockade with E2-Nb completely blocked the CDC efficacy of E2-BAR, while E3-Nb abolished the activity of E3-BAR. In contrast, CD38-STARs retained high CDC activity, demonstrating over 90% killing irrespective of E2 or E3 blockade.

The ability of CD38-STARs to overcome CD38 escape variants was further evaluated by assessing binding to HEK293T cells and CDC against CHO cells, both expressing the CD38 mutations CD38^S274F^ or CD38^K71I^ (**Figure 6**). The CD38^S274F^ mutation abrogated the binding of daratumumab (**Figure 6A**), resulting in a complete loss of CDC (**Figure 6B**). In contrast, both CD38-specific BARs and CD38-STARs maintained binding and effectively killed CD38^S274F^-expressing target cells. The predicted E2 mutation CD38^K71I^ selectively abolished both the binding (**Figure 6A**) and the cytotoxic activity of E2-BAR (**Figure 6B**). CD38-STARs, E3-BAR and daratumumab retained their binding capacity and CDC efficacy against cells expressing the CD38^K71I^ mutation.

**Figure 6:**
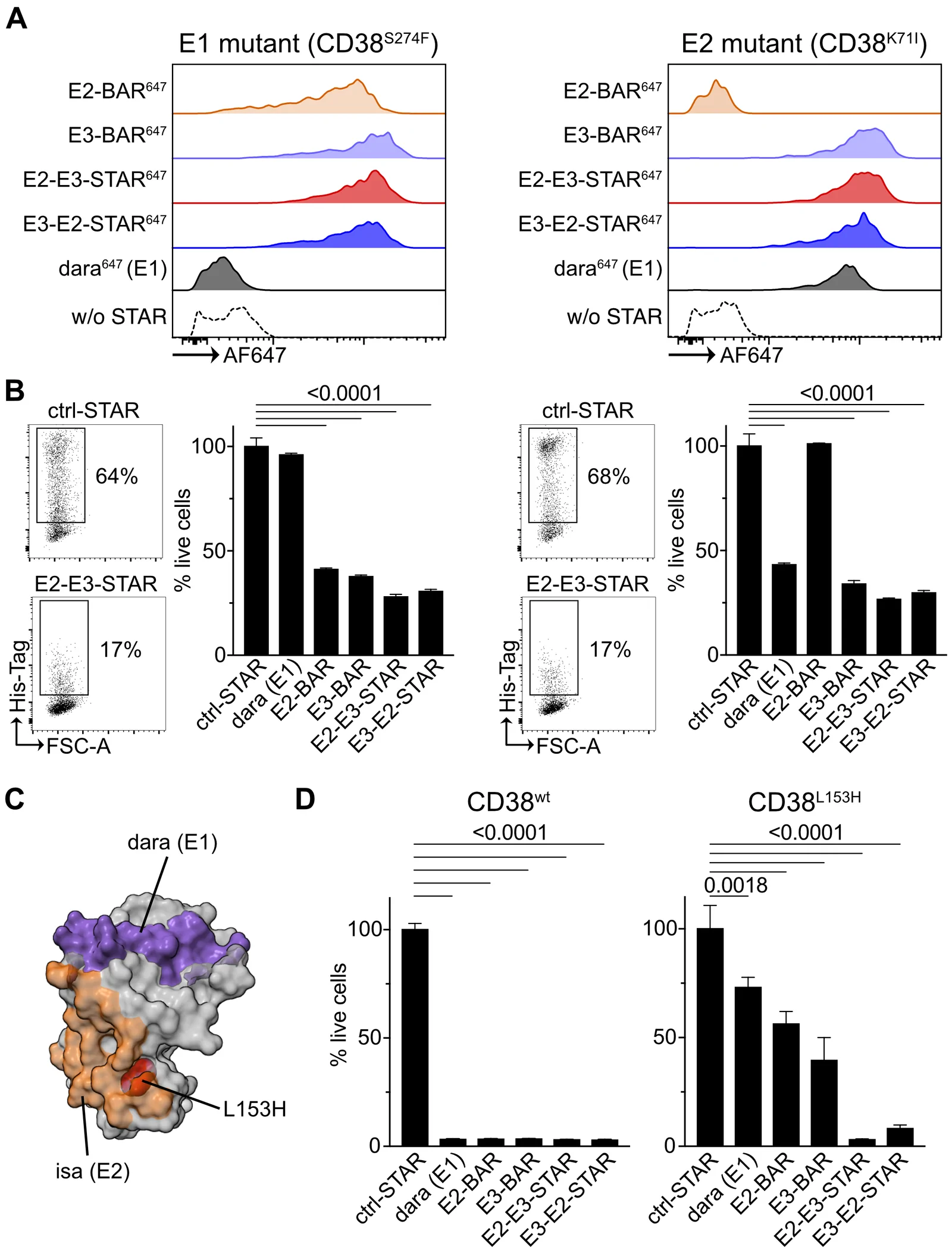
CD38-STARs retain binding and complement-dependent cytotoxicity (CDC) against cells expressing CD38 epitope mutations S274F, K71I, and L153H. **(A)** HEK293T cells were transiently transfected to express the His-tagged CD38 mutations CD38^S274F^ on epitope 1 (E1) or CD38^K71I^ on epitope 2 (E2). Cells were then stained using AF647-labelled BARs, STARs, and daratumumab. Transfected cells were detected using a His-tag-specific, FITC-labelled antibody. Gating was performed on live cells expressing His-tagged protein (not shown). **(B)** CDC assay using CHO cells expressing His-tagged CD38^S274F^ or CD38^K71I^. Representative flow cytometry plots showing the percentage of His-positive target cells following the CDC assay with ctrl-STAR or E2-E3-STAR. Quantification of target cell viability is shown next to the flow cytometry plots. Viability of His-positive cells for therapeutic constructs was normalized to ctrl-STAR (set to 100%). **(C)** Structural surface representation of the human CD38 extracellular domain (gray). The daratumumab binding epitope (dara, E1) is highlighted in purple, and the isatuximab epitope (isa, E2) is shown in orange. The location of the clinical L153H mutation is indicated in red. **(D)** CDC assay of LP-1 luc cells following CD38 knockout and subsequent transduction with wild-type CD38^wt^ or the clinical CD38^L153H^ mutant. Target cell viability was normalized to ctrl-STAR (set to 100%). Data are shown as mean ± SD of technical triplicates. Statistical significance compared to ctrl-STAR was determined using a one-way ANOVA followed by Dunnett’s multiple comparisons test, and p-values are indicated.

To translate our findings from the initial CHO cell model into a more clinically relevant MM context, we evaluated CD38-STARs against the CD38^L153H^ mutation, which is known to mediate clinical resistance to both daratumumab and isatuximab (**Figure 6C**). For these CDC assays, we utilized LP-1 luc myeloma cells with a genetic CD38 knockout, which were subsequently engineered to stably re-express high levels of either wild-type CD38 (CD38^wt^) or the CD38^L153H^ mutation (**Figure 6D**). While target cells expressing CD38^wt^ were effectively killed by all therapeutic constructs, the CD38^L153H^ mutation resulted in a substantial reduction in daratumumab-mediated killing, alongside a decrease in the cytotoxicity mediated by both CD38-specific BARs. CD38-STARs, by contrast, retained high cytotoxic efficacy against CD38^L153H^-expressing LP-1 luc cells.

### CD38-STARs mediate moderate ADCC of tumor cell lines *in vitro*

To evaluate ADCC induction, Daudi luc, CA-46 luc, and LP-1 luc cells were incubated for 3 hours with hCD16-transduced NK92 CD38KO effector cells in the presence of STARs or BARs and heat-inactivated serum as a source of immunoglobulins (**Figure 7**). Daratumumab served as a positive control and confirmed target cell sensitivity to ADCC. Both CD38-STARs induced moderate ADCC across all target cell lines, confirming their capacity to mediate antibody-dependent cellular cytotoxicity *in vitro* (**Figure 7**). CD38-specific BARs induced slightly higher ADCC efficacy than CD38-STARs.

**Figure 7:**
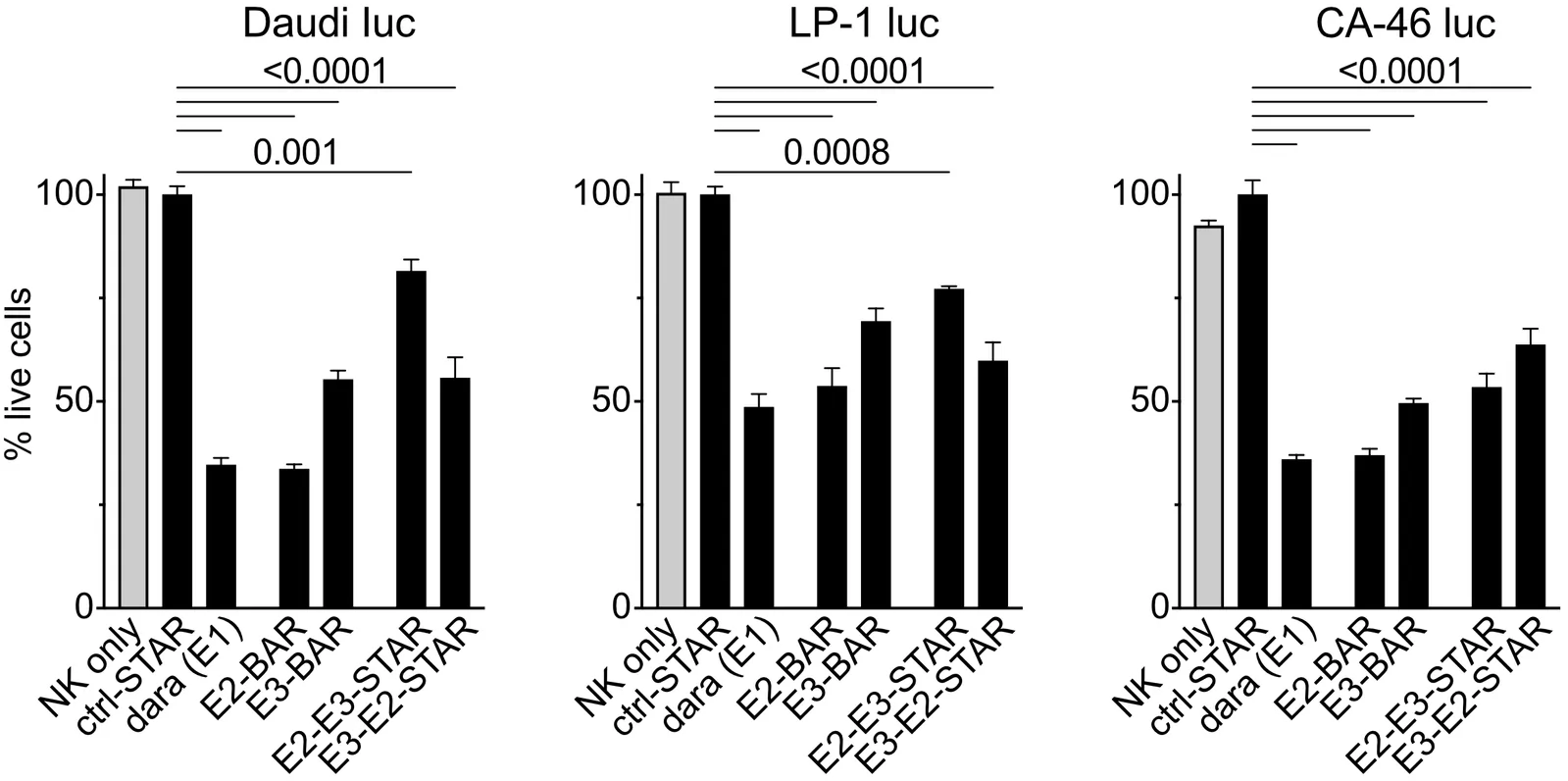
CD38-STARs induce antibody-dependent cellular cytotoxicity (ADCC) against CD38-expressing cell lines *in vitro*. Viability of Daudi luc, LP-1 luc, and CA-46 luc cells was measured after incubation with 100 nM of STARs or BARs in the presence of NK92 hCD16 CD38KO effector cells at a 3:1 effector-to-target ratio and inactivated human serum as a source of immunoglobulins for 180 min. Daratumumab (dara) was used as a positive control. Cells incubated with ctrl-STAR or without STAR (NK only) served as negative controls. Results were calculated relative to ctrl-STAR (set to 100% live cells). Data represent mean ± SD. Results of a one-way ANOVA with Dunnett’s multiple comparisons test between ctrl-STAR and samples are indicated as *p* values above the bars.

### CD38-STARs enable strong CDC against primary myeloma cells *ex vivo*

Fresh bone marrow cells from five MM patients (clinical characteristics summarized in **Supplementary Table S1**) were used to evaluate the capacity of CD38-STARs to induce CDC *ex vivo*. CD38 was expressed at high but variable levels across MM cells from all patient samples, alongside heterogeneous expression of both CD55 and CD59 (representative flow cytometry plots shown in **Supplementary Figure S1**). CDC was performed using 25% human serum as a source of complement (**Figure 8**). The non-specific ctrl-STAR was normalized to 100% viability and served as the negative control. The results show that CD38-STARs induced potent killing of MM cells within 90 min of incubation (E2-E3-STAR viability: 20.80 ± 17.66%, *p* = 0.0066; E3-E2-STAR viability: 24.60 ± 14.12%, *p* = 0.044). The positive control daratumumab also induced strong killing (viability: 14.00 ± 17.48%, *p* = 0.0014), with no statistically significant difference observed between STARs and daratumumab (both *p* > 0.05). By contrast, E2-BAR and E3-BAR mediated variable CDC efficacy (viability: 43.40 ± 23.16% and 39.80 ± 26.20%, *p* > 0.05). While E2-E3-STAR and E3-E2-STAR achieved a stronger average reduction in cell viability compared to BARs, pairwise comparisons between BARs and either STARs or daratumumab did not reach statistical significance (all *p* > 0.05).

**Figure 8:**
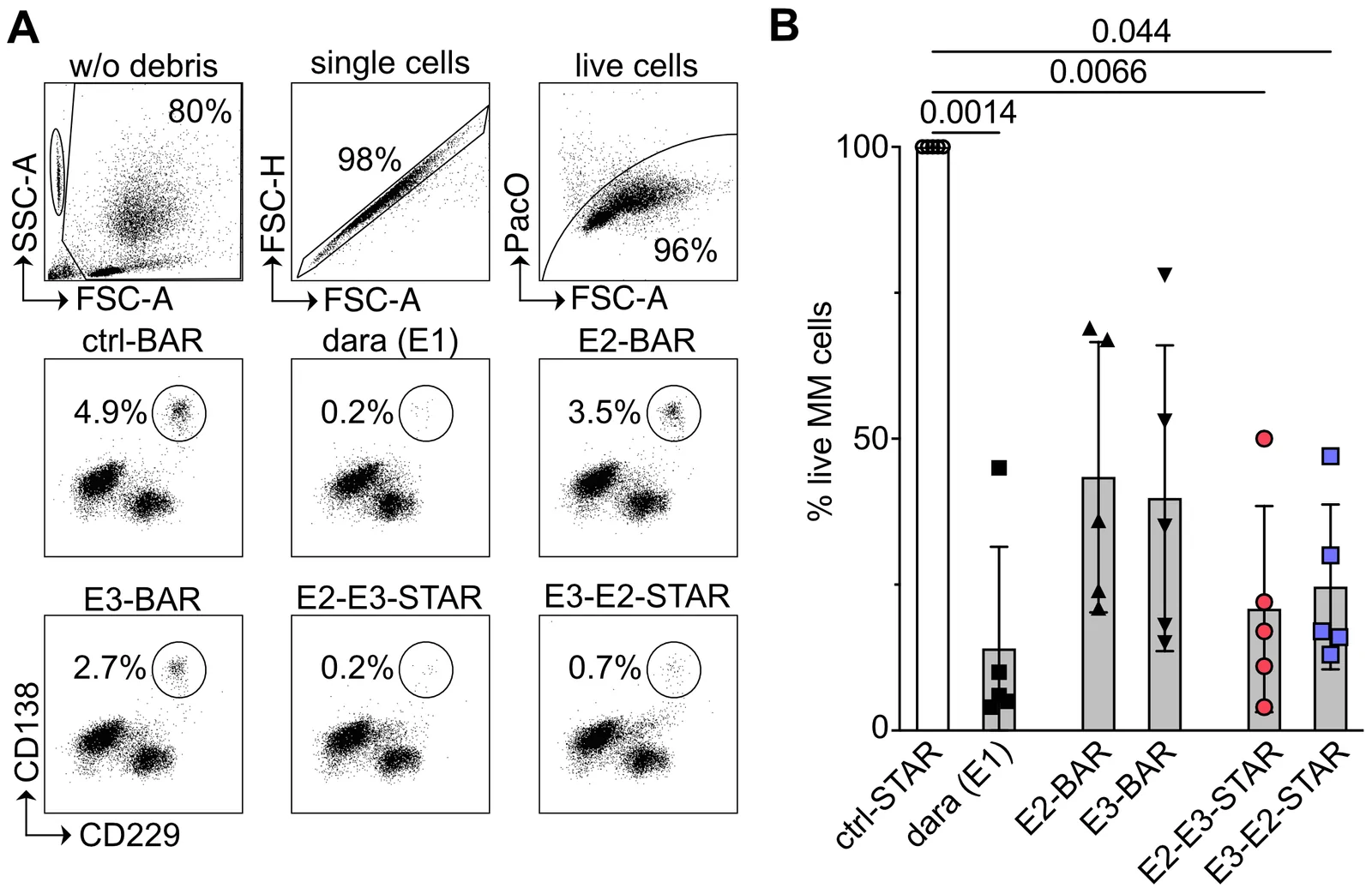
CD38-STARs induce complement-dependent cytotoxicity (CDC) against primary bone marrow cells from myeloma patient *ex vivo*. STARs and BARs were incubated with bone marrow mononuclear cells from myeloma patients (*n* = 5) in the presence of 25% human serum as a source of immunoglobulins and complement. Daratumumab (dara) was used as a positive control, a ctrl-STAR served as negative control. After 90 min, CDC was quantified by flow cytometry, using strategic staining for myeloma cells and Pacific Orange (PacO) to discriminate between live and dead cells. **(A)** Gating was performed on live cells by excluding beads, cellular debris, doublets, and dead cells (top row, left to right). The myeloma cell population was identified by high expression of both CD138 and CD229. Percentages of myeloma cells within each gate are indicated. **(B)** Cell counting beads were used to determine the absolute numbers of surviving myeloma cells. For relative quantification, the percentage of live myeloma cells was calculated relative to the ctrl-STAR (set to 100%). Data represent mean ± SD. Results of a Friedman test followed by Dunn’s multiple comparisons test between samples and the ctrl-STAR are indicated as *p* values.

CD38-STARs and daratumumab induced a significant reduction in cell viability *ex vivo*, while CD38-specific BARs did not achieve significant CDC.

In summary, CD38-STARs demonstrated high avidity, which translated into potent CDC activity even in the presence of epitope blockade or clinical CD38 mutations, as well as effective lysis of primary cells *ex vivo*.

## Discussion

Our results demonstrate the feasibility of using biparatopic CD38-STARs to improve antibody recruitment and complement-dependent killing of myeloma cells compared to monoparatopic BARs.

Both STARs displayed specific and simultaneous binding to epitopes E2 and E3 of CD38 and to the κ light chain of human serum immunoglobulins. Enhanced avidity was observed for the two STARs compared to the respective BARs and daratumumab. Cross-blockade analyses indicated a biparatopic binding mode of the two STARs. E2-E3-STAR showed stronger binding than E3-E2-STAR, characterized by slightly higher avidity and reduced susceptibility to pre-blocking. This indicates that domain arrangement is a contributing factor influencing paratope accessibility and geometric fit, in line with published reports (22, 23). Our findings suggest that positioning the higher-affinity CD38-specific nanobody in direct proximity to the κ light chain nanobody may be advantageous for optimal antigen engagement. Pre-blocking of epitope 3 led to a moderate decrease in MFI for both CD38-STARs, whereas epitope 2 blockade had no impact. This points toward epitope 3 engagement being the primary determinant of the overall binding of these constructs, likely reflecting the higher affinity of E3-Nb compared to E2-Nb (23).

The enhanced binding profile of both STARs translated into significantly more potent induction of CDC *in vitro* compared to monoparatopic BARs and daratumumab. *Ex vivo*, CD38-STARs and daratumumab induced significant lysis of primary human myeloma cells, whereas CD38-specific BARs failed to achieve significant CDC. Across the different cell lines tested, the potent CDC induction by STARs was preserved even at lower cell surface levels of CD38, whereas CDC induction by daratumumab decreased. This may allow STARs to induce effective CDC even when CD38 is downregulated. The E2-E3-STAR configuration demonstrated modestly higher CDC activity compared to E3-E2-STAR, possibly reflecting its superior binding capacity.

These results build upon our earlier findings demonstrating enhanced CDC induction by a combination of two BARs targeting distinct CD38 epitopes compared to the respective individual BARs alone (18). Improved CDC induction by biparatopic antibody constructs was previously described for several other therapeutic constructs, including CD38-specific heavy chain antibodies (19) and HER2-specific conventional antibodies (37). This effect is primarily attributed to improved avidity and cross-linking, which facilitate increased receptor clustering and thus IgG hexamerization on the cell surface (19, 37–40).

Of note, the biparatopic architecture of STARs containing E2- and E3-specific nanobodies allows these STARs to bind to MM cells treated with E1-specific daratumumab. Thus, these STARs represent a promising novel therapeutic approach in CD38-directed immunotherapy. Given that daratumumab treatment is shown to result in sustained blocking of its targeted epitope (36), the independent binding of our STARs offers a rationale for synergistic therapeutic strategies. The presence of BARs or STARs did not impair daratumumab-mediated cytotoxicity but instead achieved enhanced myeloma cell killing. This synergistic interaction suggests promising clinical potential for combination regimens or sequential use of CD38-STARs in patients progressing after daratumumab therapy. However, potential cumulative toxicities associated with dual therapies warrant careful clinical evaluation.

Additionally, the biparatopic design demonstrated functional resilience to epitope loss (13), ensuring that the compromise of a single binding site does not lead to a loss of target engagement. CD38-STARs remain functional even when the daratumumab epitope is inaccessible, as demonstrated by their sustained killing capacity against the CD38^S274F^ and the clinical CD38^L153H^ mutations.

As this approach cannot overcome resistance mechanisms driven by the complete biallelic loss of CD38 (13), the development of bispecific constructs targeting distinct tumor antigens remains a strategic option for future research.

In contrast to complement-dependent cytotoxicity, the biparatopic STAR architecture did not improve ADCC. This is in line with our previous findings demonstrating that combining two single BARs targeting distinct CD38 epitopes improved CDC but not ADCC (18). Lower antibody densities are required to reach the activation threshold for ADCC than for CDC (41, 42). Given that monoparatopic CD38-specific BARs induced ADCC at levels comparable to daratumumab, it is reasonable to assume that their antibody recruitment is sufficient to reach the ADCC activation threshold. Any further increase in avidity achieved by STARs would thus provide no additional benefit. Additionally, low- or intermediate-affinity binders allow for a higher local density of Fc domains, which is attributed to dynamic, monovalent interactions that favor ADCC induction (40). In contrast, the rigid steric cross-linking inherent to the biparatopic architecture may restrict the molecular mobility necessary to establish effective third-order avidity interactions with Fcγ receptors (40). It is conceivable that this structural rigidity could potentially result in an unfavorable orientation of the recruited antibody Fc domains, altered antibody spacing on the cell surface, and reduced overall Fc accessibility, thereby preventing further enhancement of ADCC (43).

As previously highlighted, the reliance on the κ light chain introduces a potential limitation due to competitive inhibition by elevated free light chains in a subset of myeloma patients (18). Nevertheless, this design offers advantages over conventional mAbs, e.g., recruitment of diverse immunoglobulin isotypes (IgG, IgM, IgA). Consequently, this mechanism leverages the full endogenous antibody repertoire, potentially overcoming limitations of monoclonal IgG-based therapies (44, 45). The strong CDC efficacy mediated by the STAR constructs may result from several complementary mechanisms. Recruitment of IgM could facilitate efficient complement activation via the classical complement pathway (45). Alternatively, or in addition, we hypothesize that the predicted high density of IgG recruited to the cell surface may promote hexamerization and subsequent C1q recruitment (39, 46). Nanobody-based therapeutics often employ half-life extension strategies to avoid the rapid renal excretion typically associated with low molecular weight constructs (22, 47, 48). Since binding to immunoglobulins via the κ light chain has been shown to significantly extend half-life of BARs *in vivo* (18), it is anticipated that this mechanism also applies to STARs.

Unlike conventional therapeutic antibodies, STARs lack an intrinsic Fc domain. Consequently, the induction of cytotoxicity is entirely dependent on the Fc domains of the recruited endogenous immunoglobulins. This design prevents non-specific, off-target immune activation while the molecules are freely circulating in the bloodstream, ensuring that Fc-mediated effector functions are only triggered upon specific binding to the CD38 target antigen (49). The high homology of nanobodies to human VH genes ensures minimal immunogenicity, reducing the risk of adverse immune events despite their camelid origin (16).

However, the recruitment of diverse endogenous immunoglobulin subclasses also introduces the potential risk of an excessive complement response, leading to systemic immune activation (50). On-target, off-tumor toxicities may occur due to baseline CD38 expression on normal tissues and cells, such as plasma cells, NK cells, and activated T cells (51). The tolerability and toxicity of CD38-STARs warrant careful evaluation in future studies.

We acknowledge several further limitations in the current study. Detailed mechanistic studies directly quantifying CDC dynamics, IgG density, and the impact of steric constraints or varying effector-to-target ratios on ADCC efficacy have not yet been performed and remain a focus for future research. The potential interference of soluble free κ light chains acting as a competitive ‘sink’ for CD38-STARs requires more detailed investigation. To confirm CDC efficacy of CD38-STARs at lower CD38 expression across a controlled antigen-density range, an isogenic cell system will be needed to account for intrinsic differences such as the expression of complement-inhibiting factors. Our current analyses are based on a limited patient cohort, precluding broad conclusions. Lastly, future *in vivo* studies are warranted to assess pharmacokinetics, biodistribution, toxicity, and therapeutic efficacy of CD38-STARs.

While daratumumab remains a clinical benchmark (1), CD38-STARs target a distinct therapeutic niche within the evolving multiple myeloma landscape. The biparatopic nature of STARs provides a critical structural advantage against target downregulation and single-epitope loss. Despite the remarkable efficacy of multi-specific immune engagers and cellular therapies (including CAR-T cells and BCMA-targeted approaches) (6–10), they are frequently limited by a strict reliance on T-cell fitness (6, 52). As these engagers typically lack the ability to induce CDC due to suppression of Fc-mediated effector functions (53), the complement system might represent a relevant rescue mechanism in this context.

In conclusion, our results demonstrate the feasibility of using biparatopic CD38-STARs to improve complement-mediated killing of myeloma cells over monoparatopic BARs.

## Data Availability

The original contributions presented in the study are included in the article/**Supplementary Material**. Further inquiries can be directed to the corresponding author.
