# Supplementary material for: Biparatopic targeting of CD38 using CD38-specific trimeric antibody recruiters improves cytotoxicity against multiple myeloma
Source: Front Immunol. 2026 Sep 24;17:1915205. doi: 10.3389/fimmu.2026.1915205 (PMC13646121; doi:10.3389/fimmu.2026.1915205)
Supplement: Supplementary file 1 [file SupplementaryFile1.docx]

Supplementary Material

**Supplementary Table S1. Clinical characteristics of multiple myeloma patients (*n* = 5).**

| **Clinical characteristics** | **Pat. 1** | **Pat. 2** | **Pat. 3** | **Pat. 4** | **Pat. 5** |
| --- | --- | --- | --- | --- | --- |
| Age | 56 | 60 | 74 | 69 | 65 |
| Sex | male | female | male | male | male |
| Disease stage (R-ISS) | R-ISS I | R-ISS I | n.a. | R-ISS III | R-ISS II |
| Treatment status | ND | ND | ND | R/R | ND |
| Previous anti-CD38 therapy | no | no | no | yes | no |
| Cytogenetic risk | normal | normal | normal | HR | normal |
| Immunoglobulin subtype | IgGκ | fLCκ | IgGλ | flCλ | fLCκ |
| Median serum free light chain | 725 mg/L | 1721 mg/L | 175 mg/L | 1048 mg/L | 360 mg/L |
| **Bone marrow analysis** |  |  |  |  |  |
| Infiltration rate (%) | 11.3% | 4.8% | 8.1% | 7.2% | 56.1% |
| CD38 ratio (MM / Non-MM) | 11.10 | 12.64 | 64.60 | 4.56 | 5.45 |
| CD55 ratio (MM / Non-MM) | 3.65 | 0.63 | 3.10 | 0.69 | 3.68 |
| CD59 ratio (MM / Non-MM) | 6.48 | 1.06 | 3.46 | 1.37 | 0.83 |

Expression ratios for CD38, CD55, and CD59 were calculated by dividing the median fluorescence intensity (MFI) of MM cells by the MFI of Non-MM cells. **Abbreviations:** **fLC,** free light chain; **HR,** high risk; **MM,** multiple myeloma; **n.a.,** not available; **ND,** newly diagnosed; **R/R,** relapsed/refractory; **R-ISS,** Revised International Staging System.

**Supplementary Figure 1. Flow cytometry analysis of primary bone marrow samples from multiple myeloma patients.** Representative flow cytometry dot plots characterizing bone marrow aspirates from five multiple myeloma patients (Pat. 1 – Pat. 5). The top row displays the expression of CD38 and CD138. Multiple myeloma (MM) cell population is highlighted in red (circled). Non-malignant bone marrow cells are shown in black. Percentages indicate the infiltration rate of malignant MM cells within the respective samples. The middle and bottom rows show the expression levels of the complement regulatory proteins CD55 and CD59 plotted against CD38 expression. The color coding (red for MM cells, black for non-MM cells) corresponds to the populations defined in the top row. Median fluorescence intensity (MFI) values derived from these analyses were used to calculate the expression ratios detailed in Supplementary Table S1.
